# *Lacticaseibacillus rhamnosus* B6 alleviates metabolic dysfunction-associated fatty liver disease by suppressing intestinal LPS synthesis and regulating lipid metabolism

**DOI:** 10.3389/fendo.2026.1755982

**Published:** 2026-02-16

**Authors:** Danqi Wang, Jin Han, Xiaohua Wang, Jing Wang, Chunping You, Zhengjun Wu

**Affiliations:** State Key Laboratory of Dairy Biotechnology, Shanghai Engineering Research Center of Dairy Biotechnology, Dairy Research Institute, Bright Dairy & Food Co., Ltd., Shanghai, China

**Keywords:** gut-liver axis, *Lacticaseibacillus rhamnosus*, LPS, metabolic dysfunction-associated fatty liver disease, microbiome

## Abstract

**Introduction:**

Metabolic dysfunction-associated fatty liver disease (MAFLD) has become a global epidemic with an unclear etiology and no effective therapeutic options. Disruption of the gut–liver axis driven by intestinal dysbiosis is closely implicated in MAFLD pathogenesis, making gut microbiota-targeted probiotic interventions promising preventive strategies.

**Methods:**

*Lacticaseibacillus rhamnosus* B6, a probiotic strain isolated from homemade Bulgarian fermented milk, synthesizes immunomodulatory macromolecules and regulates the intestinal flora. In the present study, we comprehensively investigated the colonization ability and MAFLD-alleviating effects of *L. rhamnosus* B6 in a high-fat diet (HFD)-induced murine MAFLD model using an integrated approach encompassing metagenomics, untargeted metabolomics, serum biochemical assays, and liver histopathological analysis.

**Results:**

Supplementation with *L. rhamnosus* B6 markedly decreased the relative abundance of *Cupriavidus*, Desulfovibrionaceae, and Enterobacteriacea, and inhibited the predicted lipopolysaccharide (LPS) synthesis pathway, thereby suppressing the inflammatory response. Furthermore, *L. rhamnosus* B6 intervention elevated unsaturated fatty acid levels by modulating lipid metabolic pathways, specifically mitochondrial β-oxidation of long-chain saturated fatty acids, α-linolenic acid, linoleic acid, and sphingolipid metabolism, while downregulating predicted myo-inositol degradation pathways, collectively contributing to MAFLD alleviation. *In vitro*, the metabolites of *L. rhamnosus* B6 exerted potent inhibitory activity against LPS-producing bacteria (e.g., *Escherichia coli* and *Salmonella enterica*).

**Discussion:**

These findings demonstrate that *L. rhamnosus* B6 is a promising probiotic for MAFLD alleviation via dual mechanisms of attenuating inflammation and regulating lipid metabolism. This study provides compelling evidence for the specific protective effects of *L. rhamnosus* B6 against MAFLD and offers a novel probiotic-based therapeutic strategy for MAFLD.

## 1 Introduction

Metabolic dysfunction-associated fatty liver disease (MAFLD), formerly known as nonalcoholic fatty liver disease (NAFLD), is a dysmetabolic disorder characterized by a spectrum of hepatic abnormalities and is the hepatic manifestation of systemic metabolic syndrome (1, 2). Currently, nearly one-third of the global population is affected by MAFLD, and its prevalence has been steadily rising over the past few decades. This condition not only impairs the quality of life of patients but also imposes a substantial economic burden on healthcare systems (3). Despite extensive research on MAFLD pathogenesis, no theory has fully elucidated the multifactorial mechanisms underlying its development. To date, no definitive pharmacotherapies for MAFLD exist, and lifestyle interventions remain the only mainstream management strategy (4). Thus, greater awareness of the health impacts of MAFLD and the development of effective therapeutic strategies are urgently warranted.

The “multiple-hit” hypothesis proposes that gut–liver axis dysfunction induced by intestinal dysbiosis plays a crucial role in metabolic inflammation, which initiates hepatic steatosis and contributes to the entire pathological progression of MAFLD (5). Accumulating evidence highlights the significance of intestinal homeostasis in liver health, with gut–liver axis dysfunction closely linked to MAFLD pathogenesis (6–8). Consequently, the gut microbiota has emerged as a novel therapeutic target for MAFLD, and probiotic supplementation is recognized as one of the most effective strategies for restoring gut microbiome homeostasis.

Inflammation and pathological lipid accumulation in hepatocytes are the core pathological features of MAFLD. A growing body of research has confirmed that probiotics can ameliorate MAFLD by alleviating inflammation and hepatic steatosis in both animal models and clinical studies (9–12). However, studies simultaneously assessing the therapeutic efficacy of probiotics against MAFLD and elucidating the correlations between the gut microbiome, inflammation, and lipid metabolism are scarce.

*L. rhamnosus* is one of the most extensively utilized probiotics in the food industry. Specific strains of this species, such as *L. rhamnosus* GG, have been reported to protect obese mice against HFD-induced dyslipidemia (13), modulate the intestinal microbiota of patients with cirrhosis (14), and exert anti-inflammatory effects on metabolic dysfunction-associated steatohepatitis (MASH) by upregulating intestinal tight junction protein expression (15). Additionally, *L. rhamnosus* TCELL modulates gut microbiota composition and short-chain fatty acid (SCFA) profiles in HFD-fed rats (16), whereas *L. rhamnosus* LRa05 ameliorates lipid accumulation and enhances hepatic carbohydrate metabolism in HFD-fed mice (17).

In our previous study, *L. rhamnosus* B6 (CGMCC 13310), isolated from homemade Bulgarian fermented milk, exhibited potential for improving gut health, including the *in vitro* synthesis of immunomodulatory exopolysaccharides (18) and the release of multiple bioactive peptides in co-fermented milk (19, 20). This strain also demonstrates excellent technological properties, particularly freeze resistance (21). In the present study, we aimed to evaluate the colonization ability of *L. rhamnosus* B6 and its comprehensive efficacy in alleviating MAFLD using an integrated approach combining 16S metagenomics and untargeted metabolomics, while clarifying the underlying mechanisms by which *L. rhamnosus* B6 acts as a probiotic in the gut–liver axis.

## 2 Methods

### 2.1 Bacterial strain and reagents

*L. rhamnosus* strain B6 (CGMCC 13310) was provided by the State Key Laboratory of Dairy Biotechnology (Shanghai, China). The strain was routinely maintained on deMan–Rogosa–Sharpe (MRS) agar plates (Thermo Scientific™) and preserved long-term in MRS broth supplemented with 20% glycerol at −80 °C. Prior to the gavage experiments, the strain was anaerobically activated on MRS agar plates at 37 °C for 48 h in a Bugbox anaerobic workstation (Ruskinn, England) under an atmosphere of N_2_:H_2_:CO_2_ = 80:10:10. Freshly activated B6 cultures were inoculated into sterile MRS broth and incubated at 37 °C for 18 h. *L. rhamnosus* B6 cells were harvested by centrifugation at 7,500×*g* and 4 °C for 15 min, washed, and resuspended in sterile 0.9% saline to a final concentration of 1.0 × 10^8^ colony-forming units (CFU) per milliliter. Viable cell counts were determined by spread plating serially diluted aliquots onto MRS agar plates, followed by anaerobic incubation at 37 °C for 48 h.

*Escherichia coli* BNCC307544 (ATCC 43895) was purchased from BeNa Culture Collection (Henan, China). *E. coli* SJTUF40005, *Salmonella enterica* SJTUF10458, and *S. enterica* SJTUF10464 were provided by Shanghai Jiao Tong University (Shanghai, China). All strains were cultured individually on trypticase phytone yeast extract (TPY) agar plates (Thermo Fisher Scientific™) at 37 °C under aerobic conditions for 24 h.

### 2.2 Inhibition zone assay

Indicator bacterial suspensions were diluted with sterile water to 10^7^ CFU/mL, and 30 μL of each suspension was spread on TPY agar plates. The cell-free culture supernatant of *L. rhamnosus* B6 was collected by centrifugation at 12,000 rpm for 10 min, followed by boiling for 3 min to inactivate enzymes and eliminate H_2_O_2_. The antibacterial activity against LPS-producing bacteria was determined using the Oxford cup diffusion method. Briefly, the Oxford cups were placed on indicator agar plates, 100 μL of the *L. rhamnosus* B6 cell-free supernatant was added to each cup, and the plates were incubated aerobically at 37 °C for 48 h. The diameters of the inhibition zones were measured using a Vernier caliper.

### 2.3 Animal experimental design

For the colonization experiment, following a 1-week acclimation period, 20 6-week-old male C57BL/6J mice (purchased from Chengqin Biotechnology Co., Ltd., Shanghai, China) were randomly divided into two groups. All mice received a 3-day antibiotic regimen (daily gavage of 0.2 mL of 25 mg/mL ampicillin solution, equivalent to 5 mg/mouse). Subsequent to ampicillin treatment, the mice were orally administered 0.2 mL of *L. rhamnosus* B6 suspension or 0.9% sterile saline via gavage once daily for 2 weeks. Fecal samples were collected at baseline and at the 1st and 2nd week post-interventions. At the end of the gavage period, five mice per group were euthanized via carbon dioxide (CO_2_) asphyxiation in a 40 L euthanasia chamber (gas flow rate: 12 L/min), and the intestinal segments were harvested for further analysis. The microbial community composition was determined using 16S rRNA gene sequencing.

For the HFD-induced MAFLD animal experiment, 18 eight-week-old male C57BL/6J mice (Chengqin Biotechnology Co., Ltd.) were acclimated for 1 week and then randomly allocated to three groups (n = 6 per group): (1) negative control (SD group, standard diet; Research Diets, D12492i); (2) model control (HFD group, high-fat diet; Research Diets, D12450J); (3) HFD + *L. rhamnosus* B6 (HFD + B6 group, HFD plus daily gavage of 10^9^ CFU *L. rhamnosus* B6). Body weights and food intakes were recorded weekly. At the end of the 14-week study, the mice were euthanized via CO_2_ asphyxiation. Blood samples were collected by cardiac puncture into plasma separation tube. Serum was harvested by centrifugation at 4,000 rpm for 15 min at 4 °C and stored at −80 °C. Subcutaneous adipose tissue, livers, and colons were collected, weighed, and snap-frozen at −80 °C.

### 2.4 Histological analysis

Liver tissues were processed for hematoxylin and eosin (H&E) staining and scanning electron microscopy (SEM). For H&E staining, the tissues were fixed in 4% paraformaldehyde for 24 h, embedded in paraffin, sectioned into 5 μm slices, dewaxed, rehydrated, stained with H&E, and observed under a light microscope (Olympus). For SEM, liver tissues were perfused with a fixative at 4 °C for 4 h, post-fixed in 0.1% osmium tetroxide at room temperature for 2 h, rinsed with PBS, dehydrated, embedded, and sectioned into 60 nm–80 nm ultrathin slices. The slices were stained with 2% uranyl acetate and lead citrate, air-dried overnight, and examined under a scanning electron microscope (Hitachi, Tokyo, Japan).

### 2.5 Biochemical parameters measurements

Serum total cholesterol (TCH) and low-density lipoprotein cholesterol (LDL-C) were measured using a Beckman Chemistry Analyzer AU2700 System (Beckman Coulter, Tokyo, Japan). Hepatic triglyceride (TG) levels were assayed by homogenizing liver tissues, centrifuging to collect supernatants, and using a triglyceride quantification kit (Abcam, ab65336) according to the manufacturer’s instructions.

### 2.6 Cytokine measurements

Serum levels of tumor necrosis factor (TNF)-α, interleukin (IL)-1β, and IL-10 were quantified using enzyme-linked immunosorbent assay (ELISA) (MEIMIAN, Jiangsu, China), following the manufacturer’s instructions.

### 2.7 Gut microbiota profiling

Genomic DNA was extracted from murine colon contents and fresh fecal samples using the OMEGA Soil DNA kit (M5635-02; Omega Bio-Tek, Norcross, GA, USA), following the manufacturer’s instructions. DNA quantity and quality were assessed using a NanoDrop NC2000 spectrophotometer (Thermo Fisher Scientific, Waltham, MA, USA) and agarose gel electrophoresis. The V3–V4 hypervariable regions of the 16S rRNA gene were amplified by polymerase chain reaction using the primers 338F (5′-ACTCCTACGGGAGGCAGCAG-3′) and 806R (5′-GGACTACHVGGGTWTCTAAT-3′). The PCR reaction contained 5 μL 5×buffer, 0.25 μL FastPfu DNA polymerase (5 U/μL), 2 μL dNTP mix (2.5 mM each), 1 μL of each primers (10 μM), 1 μL DNA template, and 14.75 μL ddH_2_O. The thermal cycling program was as follows: initial denaturation at 98 °C for 5 min; 30 cycles of denaturation at 98 °C for 30 s, annealing at 55 °C for 30 s, and extension at 72 °C for 45 s; and a final extension step at 72 °C for 5 min. PCR amplicons were purified using Vazyme VAHTS™ DNA Clean Beads (Vazyme, Nanjing, China) and quantified using the Quant-iT PicoGreen dsDNA Assay Kit (Invitrogen, Carlsbad, CA, USA). Equal amounts of purified PCR products were pooled and subjected to paired-end sequencing (2 × 250 bp) on the Illumina NovaSeq platform (NovaSeq 6000 SP Reagent Kit, 500 cycles) at Genekinder Medical Technology Co., Ltd. (Shanghai, China).

Sequence data were analyzed using QIIME2 and R software (v3.2.0). ASV-level alpha diversity (Chao1 index) was calculated based on the ASV tables in QIIME2. Beta diversity was assessed using Bray–Curtis dissimilarity and visualized using principal coordinate analysis (PCoA). Phylogenetic analysis was performed using MEGA-X, and trees were visualized using iTOL (https://itol.embl.de/). Microbial functional predictions were performed using PICRUSt2 against the MetaCyc (https://metacyc.org/) and KEGG (https://www.kegg.jp/) databases.

### 2.8 Untargeted metabolomics analyses

Untargeted metabolomics of murine fecal samples was performed using an UHPLC system (Vanquish, Thermo Fisher Scientific) coupled to an Orbitrap Exploris 120 mass spectrometer (Orbitrap MS, Thermo) with a Waters ACQUITY UPLC BEH Amide column (2.1 mm × 50 mm, 1.7 μm). Briefly, 25 mg of fecal samples were mixed with grinding beads and 0.5 mL of extraction solution (MeOH: ACN:H_2_O, 2:2:1, v/v) containing deuterated internal standards. The mixtures were vortexed for 30 s, homogenized at 35 Hz for 4 min, sonicated in an ice-water bath at 4 °C for 5 min (repeated three times), incubated at −40 °C for 1 h to precipitate proteins, and centrifuged at 12,000 rpm for 15 min at 4 °C. The supernatants were transferred to new glass vials for further analysis.

Raw data were converted to mzXML format using ProteoWizard and processed using an in-house R program based on XCMS for peak detection, extraction, alignment, and integration (22, 23). Metabolite annotation was performed using the KEGG and Human Metabolome Database (HMDB). Principal component analysis (PCA), orthogonal partial least squares discriminant analysis (OPLS-DA), pathway analysis, volcano plots, and heatmaps (annotated with fold changes and adjusted *P*-values) were generated using MetaboAnalyst 6.0 (six biological replicates per group).

### 2.9 Real-time PCR analysis

Total RNA was extracted from the liver samples using TRIzol reagent (T9108, Takara) following the manufacturer’s protocol. RNA concentrations were measured using a Nanodrop analyzer (Thermo Fisher Scientific), and the extracted RNA was reverse-transcribed into complementary DNA using the PrimeScript RT reagent kit (RR037A, Takara). Real-time PCR was performed on an Applied Biosystems™ 7500 (Thermo Fisher Scientific) with gene-specific primers (**Supplementary Table S1**). The results were normalized against the housekeeping gene *GAPDH*, and the data were analyzed using the 2^−ΔΔCt^ method.

### 2.10 Statistical analysis

Statistical analyses and graphical visualizations were performed using GraphPad Prism 10.4.1. After testing for normality and homogeneity of variances, an unpaired two-tailed Student’s t-test was employed for comparisons between the two groups. For multi-group comparisons, one-way analysis of variance (ANOVA) was conducted first, followed by Tukey’s multiple comparison test for *post hoc* analysis. Statistical significance was set at *P <*0.05.

## 3 Results

### 3.1 Colonization and intestinal flora modulation

**Figure 1:**
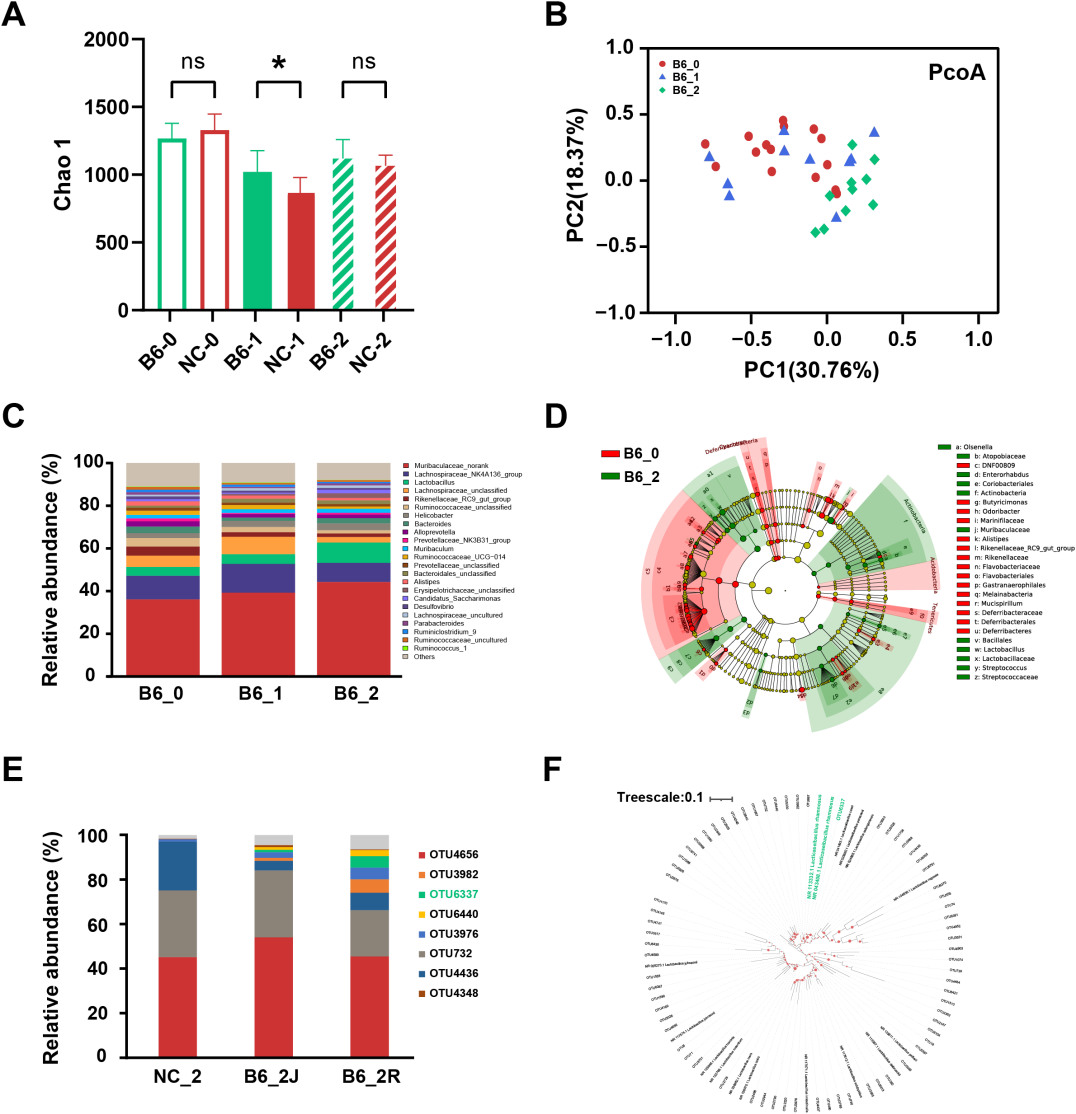
Effects of *L. rhamnosus* B6 supplementation on the intestinal microbiota in the colonization experiment. **(A)** Chao1 index of fecal samples; **(B)** PCA based on Bray–Curtis distance; **(C)** Genus-level compositional analysis of fecal samples; **(D)** LEfSe analysis; **(E)** Compositional analysis of *Lactobacillus*-related OTUs; **(F)** Phylogenetic analysis of *Lactobacillus*-related OTUs. Notes: “−0,” “−1,” and “−2” represent the study initiation, 1-week intervention, and 2-week intervention, respectively. “R” denotes the intestinal contents of mice, and “J” denotes the colon of mice. Phylogenetic analysis was executed using MEGA-X, with visualization conducted via iTOL. Statistical significance: “*” represents p <0.05, “ns” represents no significance.

Antibiotic-depleted mice were used to evaluate *L. rhamnosus* B6 colonization and the regulation of intestinal flora. Initially, fecal microbial diversity (Chao 1 index) was comparable between groups, but *L. rhamnosus* B6-supplemented mice exhibited a significantly higher Chao 1 index than controls after 1 week (*P* <0.05), though this difference diminished at 2 weeks (**Figure 1A**). These results demonstrate that *L. rhamnosus* B6 accelerates the restoration of intestinal flora after antibiotic treatment. After 2 weeks of *L. rhamnosus* B6 intervention, the gut microbiota structure became more diverse (**Figure 1B**), and the relative abundance of *Lactobacillus* increased (**Figures 1C, D**). Phylogenetic analysis of *Lactobacillus*-related OTUs confirmed that OTU6337 (16S rRNA V3-V4 sequence identical to *L. rhamnosus*) was present in the colonic and intestinal contents of mice after 2 weeks of *L. rhamnosus* B6 intervention, verifying successful colonization (**Figures 1E, F**).

### 3.2 Alleviating effect on MAFLD induced by high-fat diet

**Figure 2:**
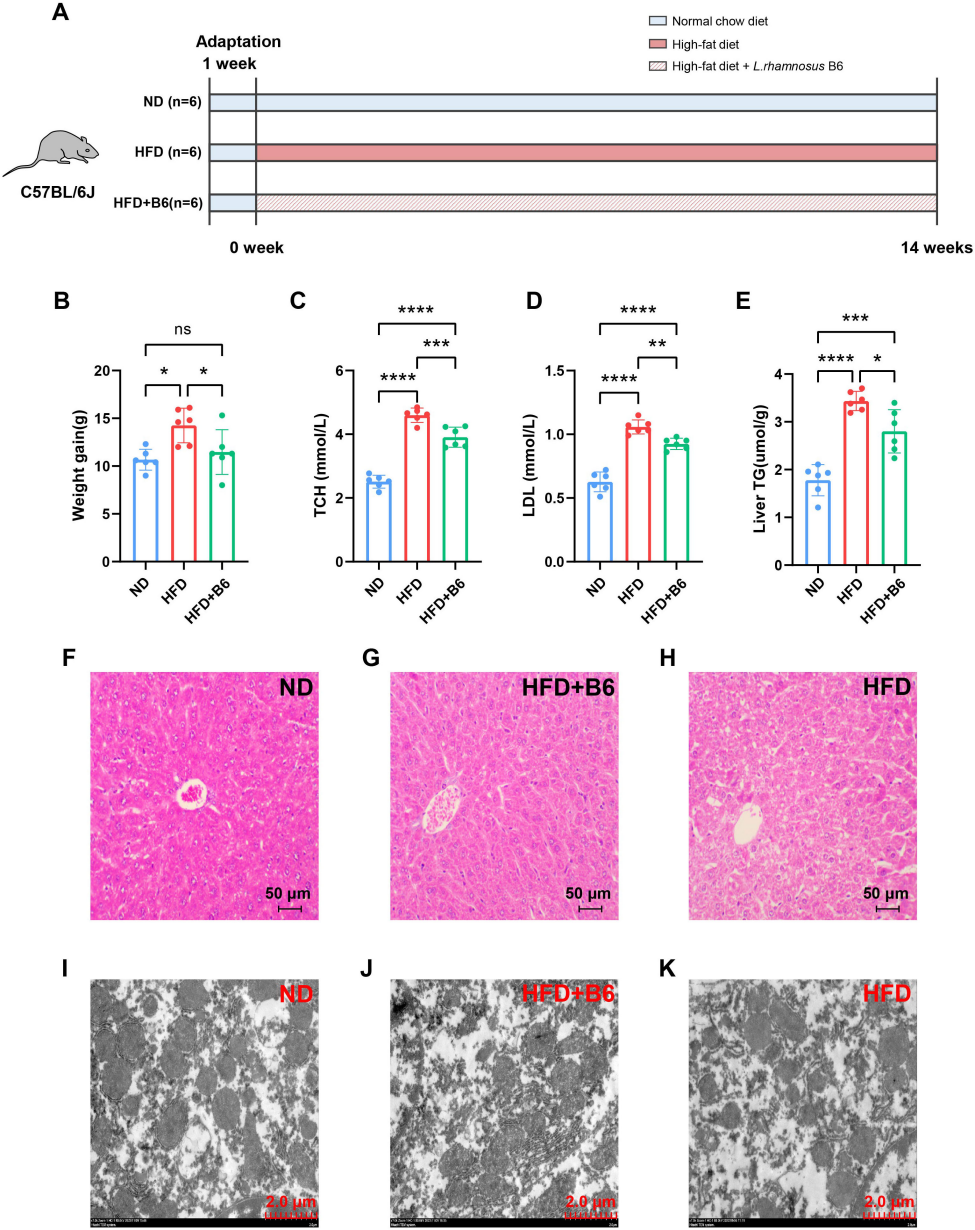
Effects of *L. rhamnosus* B6 supplementation on high-fat diet induced MAFLD mice. **(A)** Schematic of the animal experimental design; **(B)** Body weight gain; **(C)** Serum TCH levels; **(D)** Serum LDL levels; **(E)** Hepatic TG levels. Liver H&E staining of **(F)** ND group, **(G)** HFD + B6 group, and **(H)** HFD group. Liver electron micrographs of **(I)** ND group, **(J)** HFD + B6 group, and **(K)** HFD group. Statistical significance: ****p <0.0001, ***p <0.001, **p <0.01, *p <0.05.

To assess the alleviative effects of *L. rhamnosus* B6 on MAFLD, mice were fed a normal chow diet, a high-fat diet, or a high-fat diet supplemented with *L*. *rhamnosus* B6 for 14 weeks, following the experimental protocol illustrated in **Figure 2A**. Outcome indicators were measured at the end of the study. *L. rhamnosus* B6 supplementation reduced body weight gain in the HFD + B6 group to levels comparable to those observed in the ND group and significantly lower than those in the HFD group (**Figure 2B**). Regarding metabolic parameters, although serum TCH, LDL, and liver TG levels in the HFD + B6 group were higher than those in the ND group, they were markedly lower than those in the HFD group (**Figures 2C–E**). H&E staining revealed that hepatocytes in the HFD group exhibited swelling with prominent steatosis, accompanied by extensive accumulation and dispersion of lipid droplets in the cytoplasm. Mitochondrial deformation and rupture were observed, along with a disorganized rough endoplasmic reticulum. Notably, *L. rhamnosus* B6 intervention significantly ameliorated hepatocellular steatosis, reduced the number of lipid droplet vacuoles, tended to normalize mitochondrial structure, and alleviated degeneration and disorganization of the rough endoplasmic reticulum (**Figures 2F–K**). Collectively, these results demonstrate that *L. rhamnosus* B6 administration alleviates MAFLD, as corroborated by both metabolic indicators and histological observations.

### 3.3 Regulatory effect of *L. rhamnosus* B6 on inflammatory factor

**Figure 3:**
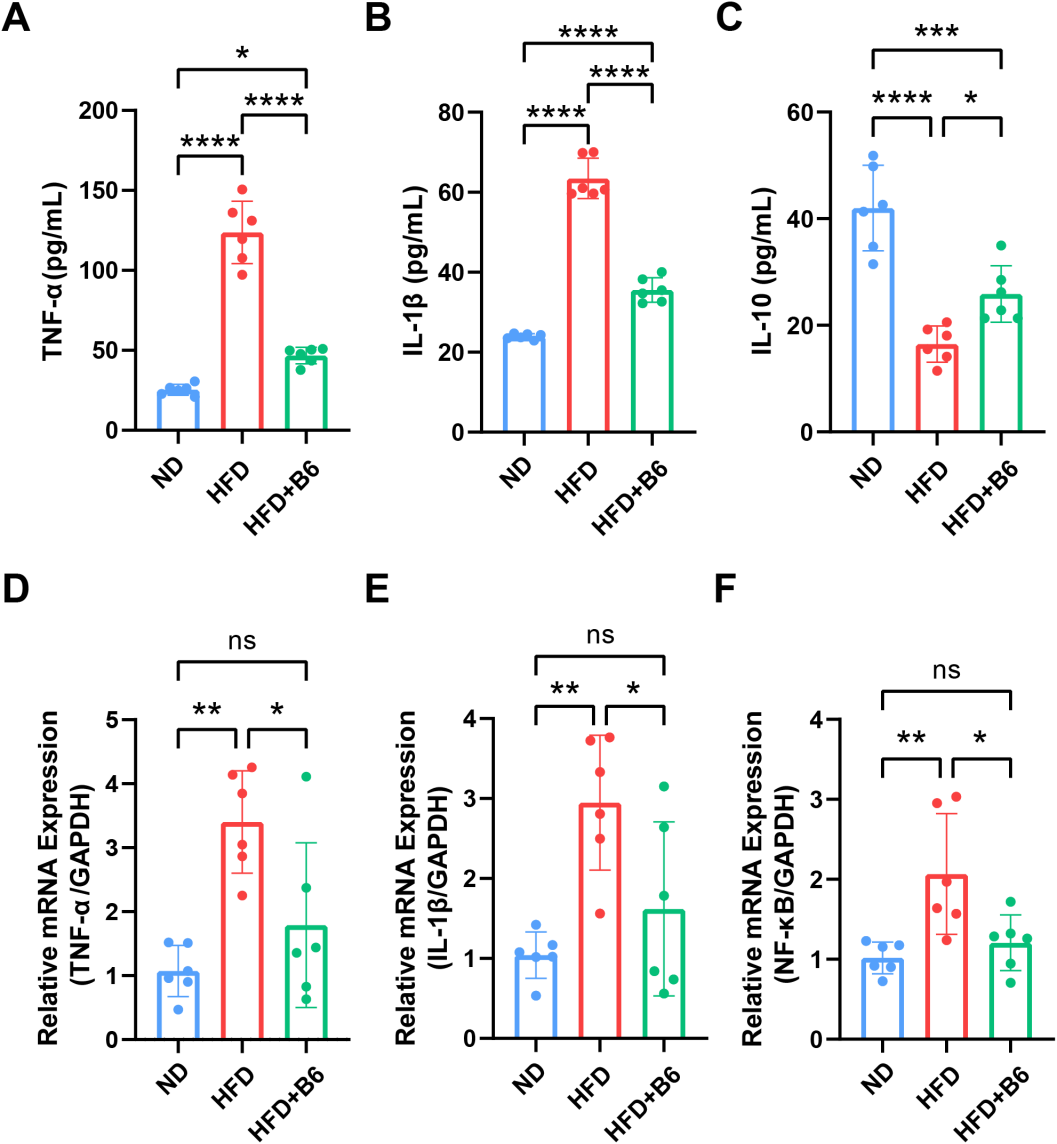
Effects of *L. rhamnosus B6* supplementation on inflammatory factors in high-fat diet induced MAFLD mice. Serum levels of **(A)** TNF-α, **(B)** IL-1β, and **(C)** IL-10. Hepatic expression levels of **(D)** TNF-α, **(E)** IL-1β, and **(F)** NF-κB. Statistical significance: ****p <0.0001, ***p <0.001, **p <0.01, *p <0.05.

Chronic inflammation is a key factor in the pathogenesis of MAFLD. The gut–liver axis, which mediates the translocation of bacterial products into the portal circulation, has been identified as an upstream trigger of inflammation in MAFLD (24, 25). Following *L*. *rhamnosus* B6 intervention, the expression of inflammatory factors in liver tissues and their concentrations in serum were determined. Compared with the HFD group, the *L. rhamnosus* B6 intervention group exhibited a marked reduction in the serum levels of the pro-inflammatory factors TNF-α and IL-1β, along with an increase in the anti-inflammatory factor IL-10, with a trend toward the levels observed in the ND group (**Figures 3A–C**). Furthermore, the expression of inflammatory factors in liver tissues was detected by RT-qPCR, which showed that *L. rhamnosus* B6 intervention significantly reduced the expression of pro-inflammatory factors TNF-α and IL-1β, as well as nuclear transcription factor NF-κB (**Figures 3D–F**). Collectively, these findings suggest that *L. rhamnosus* B6 intervention can reduce the serum concentrations of pro-inflammatory factors and subsequently suppress their expression in the liver, thereby alleviating hepatic inflammation.

### 3.4 Microbiome composition of intestinal contents

**Figure 4:**
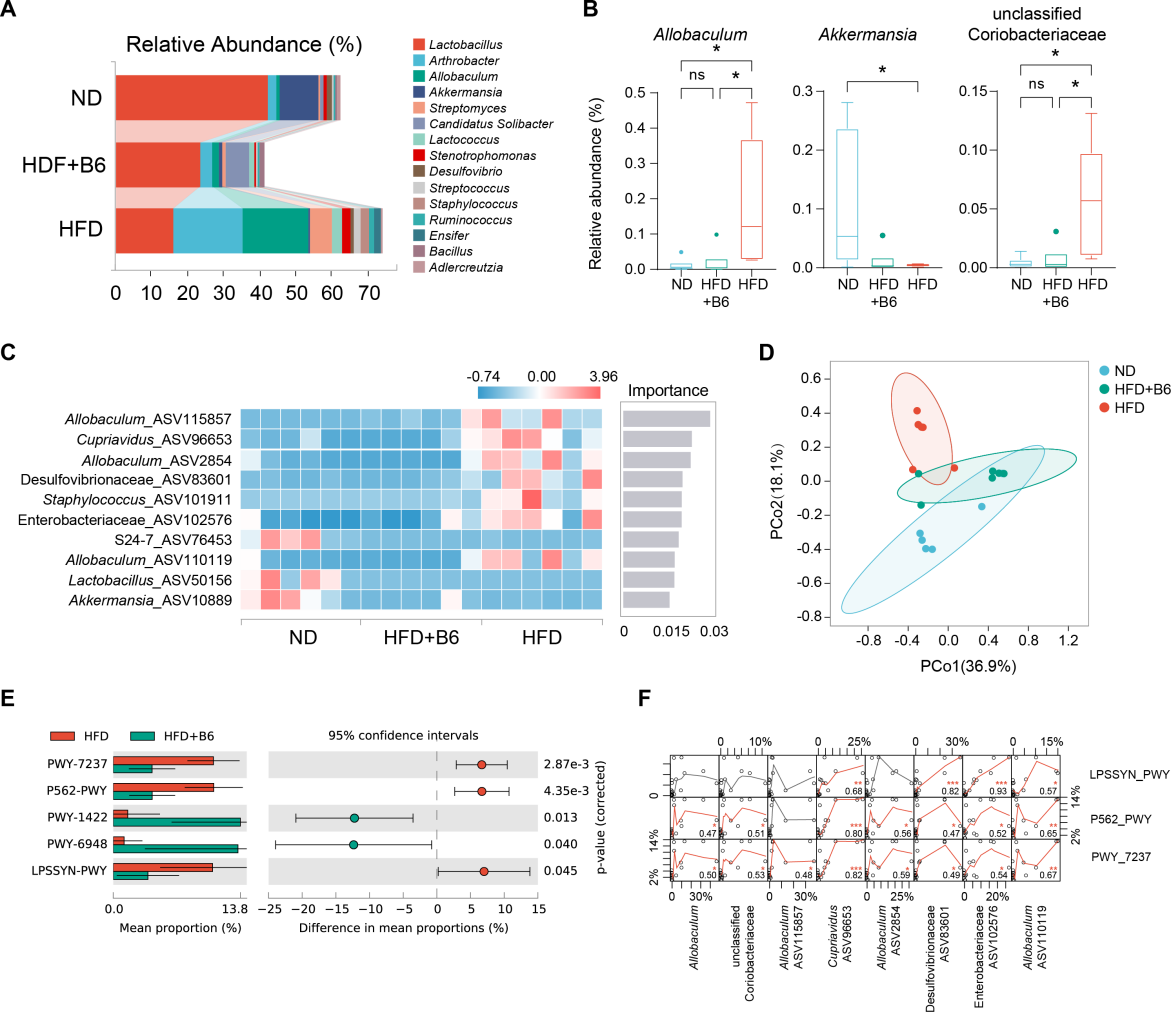
Effects of *L. rhamnosus* B6 supplementation on the intestinal microbiota in high-fat diet induced MAFLD mice. **(A)** Compositional analysis of the top 15 genera in each mouse group; **(B)** Genera with significant differences among the three groups; **(C)** Top10 ASVs with major differences among the three groups selected by random forest, sorted by importance; **(D)** PCoA analysis based on Bray–Curtis distance; **(E)** Significant different pathways predicted by PicRust2; **(F)** Correlation analysis between significantly different pathways and ASVs following *L. rhamnosus* B6 intervention. Statistical significance: ****p <0.0001, ***p <0.001, **p <0.01, *p <0.05, and “ns” represents no significance.

Accumulating evidence has demonstrated that gut microbial dysbiosis is closely associated with MAFLD progression. Therefore, intestinal contents were collected from each mouse at the end of the experiment for 16S rRNA sequencing to elucidate the underlying mechanism through which *L. rhamnosus* B6 alleviates MAFLD. At the genus level, *L. rhamnosus* B6 intervention increased the relative abundance of *Lactobacillus* compared to the HFD group, although this was not statistically significant (**Figure 4A**). Notably, *L. rhamnosus* B6 intervention significantly decreased the abundance of *Allobaculum* and an unclassified Coriobacteriaceae, while significantly increasing the abundance of *Akkermansia* (**Figure 4B**). The top 10 bacterial ASVs in each group were identified using random forest analysis and ranked by importance scores. The results indicated that *L. rhamnosus* B6 intervention suppressed the abundance of several ASVs belonging to *Allobaculum*, *Cupriavidus*, Desulfovibrionaceae, and Enterobacteriacea (**Figure 4C**). PCoA based on the Bray–Curtis dissimilarity metric revealed that the gut microbial community structure in the *L. rhamnosus* B6-treated group differed from that in the HFD group but was closer to that in the ND group, highlighting the regulatory effect of *L. rhamnosus* B6 administration on gut microbial composition (**Figure 4D**). PICRUST2 was employed to predict the microbial functional profiles based on ASV abundance. The PWY-1422 and PWY-5948 pathways were upregulated, whereas pathways involved in myo-inositol degradation (PWY-7237 and PWY-562) and LPS synthesis (LPSSYN-PWY) were downregulated following *L. rhamnosus* B6 intervention, and these profiles differed significantly from those in the HFD group (**Figure 4E**). Spearman’s correlation analysis was performed to explore the associations between ASVs and predicted functional pathways, as illustrated in **Figure 4F**. The ASVs suppressed by *L. rhamnosus* B6 exhibited a significant positive correlation with the pathways inhibited by *L. rhamnosus* B6 administration. In addition, antibacterial assays revealed that the fermentation supernatant of *L. rhamnosus* B6 exerted a significant inhibitory effect against Enterobacteriaceae strains, including *E. coli* BNCC307544, *E. coli* SJTUF40005, *S. enterica* SJTUF10458, and *S. enterica* SJTUF10464, with inhibition zone diameters of 19.7 mm, 20.9 mm, 20.1 mm, and 19.1 mm, respectively (**Supplementary Figure S1**). Overall, these findings indicate that *L. rhamnosus* B6 intervention downregulates LPS synthesis and myo-inositol degradation functions of the gut microbiota by inhibiting the proliferation of ASVs belonging to *Allobaculum*, *Cupriavidus*, Desulfovibrionaceae, and Enterobacteriaceae.

### 3.5 Regulating effect of *L. rhamnosus* B6 on lipid metabolism

**Figure 5:**
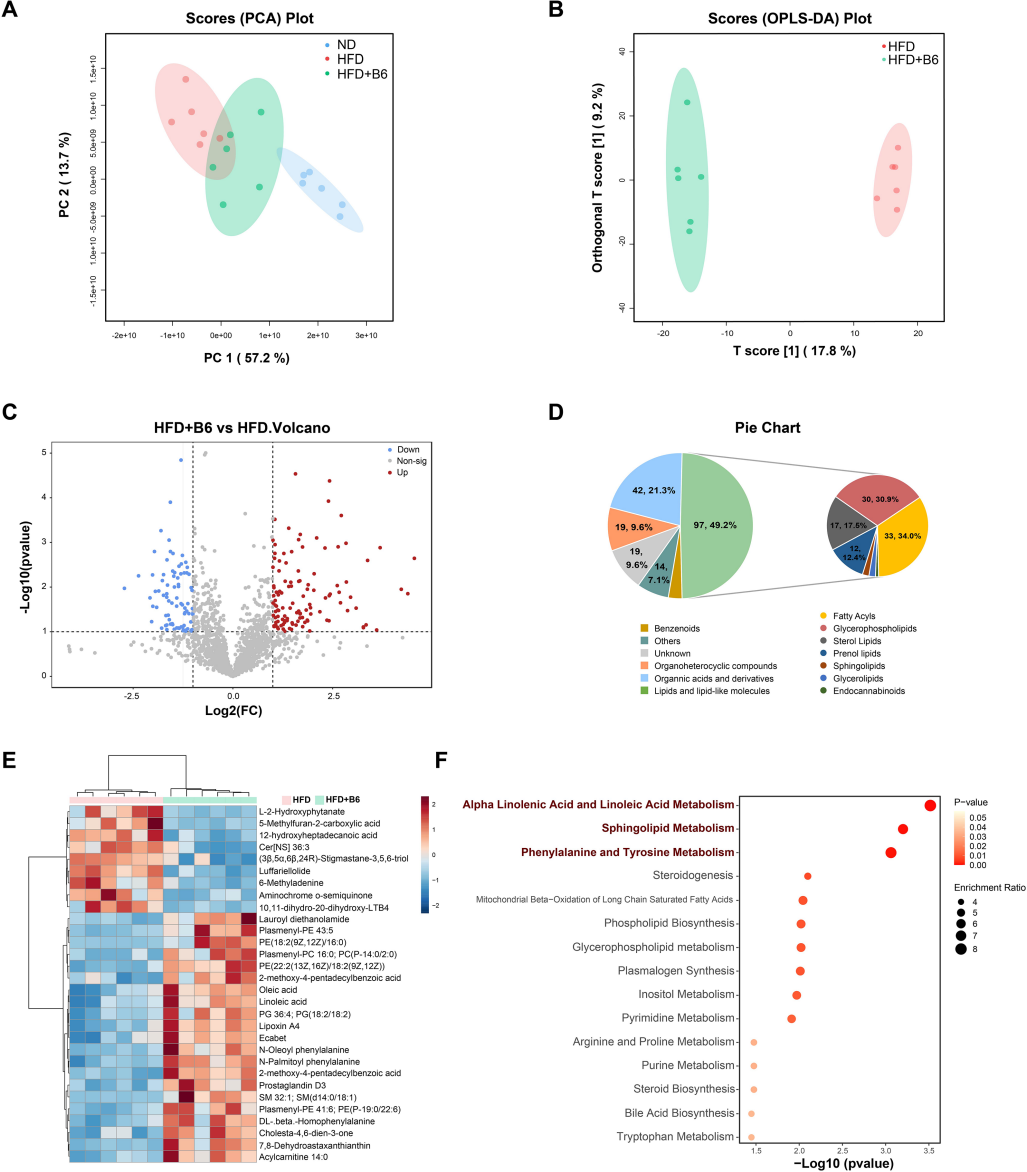
Effects of *L. rhamnosus* B6 supplementation on fecal untargeted metabolomics in high-fat diet induced MAFLD mice. **(A)** PCA plot of all three groups, represented by different colors and shapes; **(B)** OPLS-DA plot between the HFD and HFD + B6 groups; **(C)** Volcano plot between the HFD and HFD + B6 groups. Significantly altered metabolites were identified and marked in red (increased) and blue (decreased), respectively; **(D)** Classification and proportion analysis of differential metabolites annotated by Human Metabolome Database between the HFD and HFD + B6 groups. Different colors represent HMDB classifications in the pie chart, with the numbers and corresponding proportions of significantly altered metabolites listed; **(E)** Heatmap analysis of the top 30 significantly altered metabolites between the HFD and HFD + B6 groups; **(F)** KEGG pathway enrichment analysis based on significantly altered metabolites between the HFD and HFD + B6 groups. The enrichment ratio and *P* value are reflected by circle size and color, respectively.

Beyond inflammatory cytokines, the imbalance between fatty acid uptake/lipogenesis and export/metabolism leads to lipid accumulation, another key hallmark of MAFLD (26). To investigate the impact of *L. rhamnosus* B6 intervention on MAFLD-related metabolic alterations, untargeted metabolomics analysis was performed on fecal samples from mice in each group at the end of the experiment using LC-MS/MS, and the data were processed using the MetaboAnalyst platform (version 6.0). The PCA results revealed a clear separation between the three groups. Compared with the HFD group, the HFD + B6 group clustered closer to the ND group (**Figure 5A**), and the orthogonal partial least squares-discriminant analysis (OPLS-DA) score plot also displayed distinct grouping between the HFD and HFD + B6 groups (**Figure 5B**). A volcano plot was used to identify differential metabolites (DMs) between the HFD and HFD + B6 groups, and a total of 197 DMs were screened, including 116 upregulated and 81 downregulated metabolites (**Figure 5C**). These DMs were categorized into several classes, with lipids and lipid-like molecules being the most abundant, accounting for 49.2% of all significantly altered compounds modulated by *L. rhamnosus* B6. Among the differentially expressed lipids and lipid-like molecules, fatty acyls, glycophoropholipids, sterol lipids, and prenol lipids were the major components, representing 34.0%, 30.9%, 17.5%, and 12.4%, respectively (**Figure 5D**). A heatmap depicting the hierarchical clustering results of the top 30 DMs showed that nine and 21 metabolites were significantly enriched in the HFD and HFD + B6 groups, respectively (**Figure 5E**; **Supplementary Table S2**). Specifically, oleic acid, linoleic acid, and lipoxin A4 levels were elevated, whereas Cer[NS] 36:3 levels were reduced following *L. rhamnosus* B6 intervention. All DMs between the two groups were subjected to KEGG-based metabolic pathway enrichment analysis. The core metabolic pathways involving α-linolenic acid, linoleic acid, sphingolipids, phenylalanine, tyrosine metabolism, and mitochondrial β-oxidation of long-chain saturated fatty acids were identified as the core metabolic pathways associated with the ameliorative effects of *L. rhamnosus* B6 on MAFLD (**Figure 5F**).

### 3.6 Correlation profiling of ASVs, biomarkers, and metabolomics

**Figure 6:**
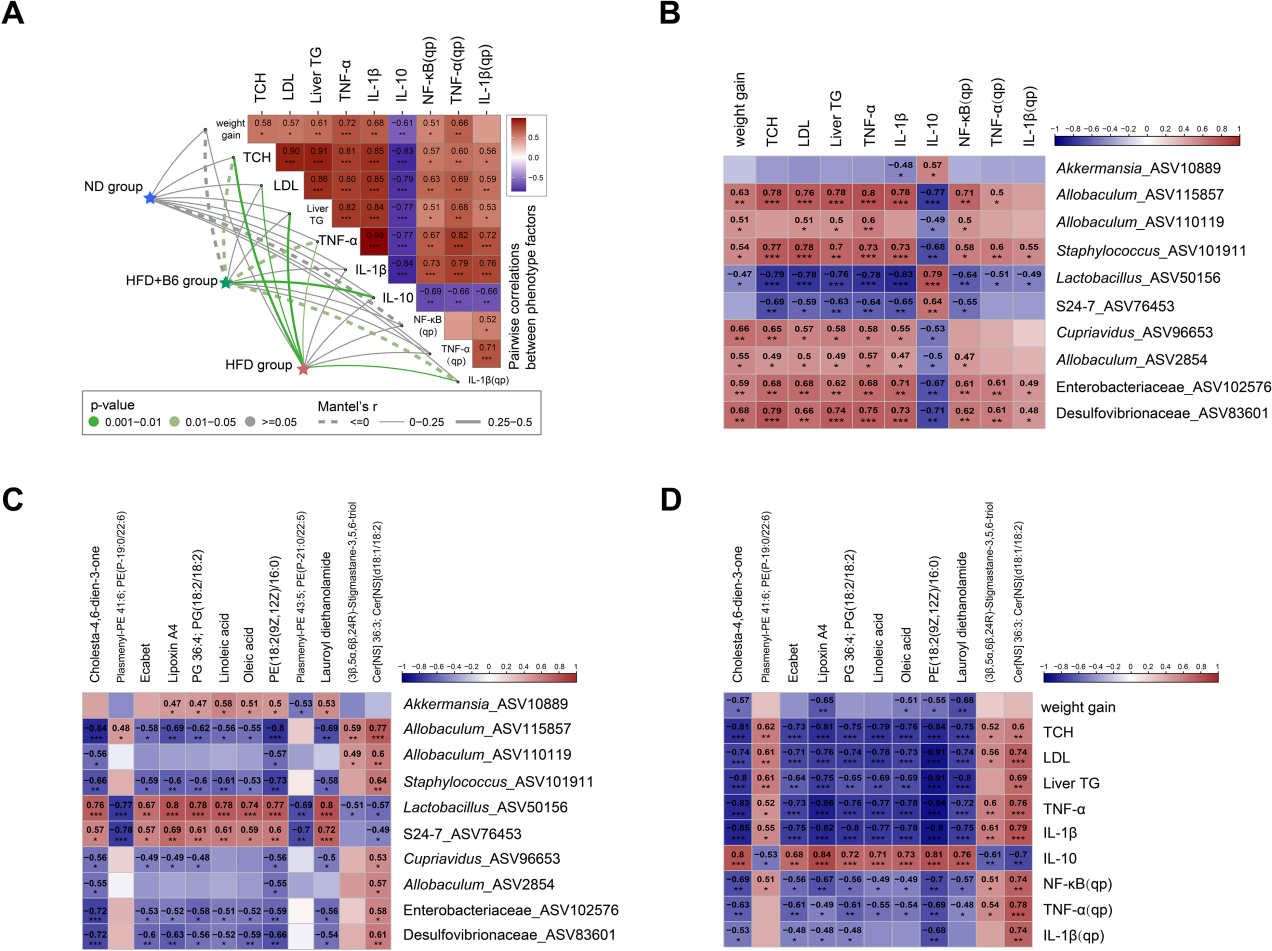
Correlations between microbial composition, blood biomarkers, and metabolites in high-fat diet induced MAFLD mice. **(A)** Pairwise comparisons of blood biomarkers and their relationships with community composition across different groups. Spearman’s correlation coefficients of blood biomarkers are displayed with a color gradient. Taxonomic community composition based on ASVs was related to blood biomarkers by partial Mantel tests. Edge type and color denote Mantel’s *r* and significance, respectively; **(B)** Associations between major discriminatory microbial ASVs and blood biomarkers; **(C)** Associations between major discriminatory microbial ASVs and 12 significantly altered lipid and lipid-like metabolite molecules; **(D)** Associations between discriminatory lipid and lipid-like metabolite molecules and blood biomarkers. Dark red and navy indicate positive and negative Spearman correlation coefficients, respectively. “*,” “**,” and “***” denote *p <*0.05, *p <*0.01, and *p <*0.001, respectively.

To further investigate the correlations among gut microbiota, metabolic biomarkers, and key DMs in MAFLD mice, a Mantel test was performed to analyze the associations between the ASVs matrix (comprising ASVs with a relative abundance >0.1%) and major biomarkers, including body weight gain, serum biochemical indices, and hepatic inflammatory cytokine expression levels. As presented in **Figure 6A**, the ASV matrix of the HFD+B6 group was negatively correlated with body weight gain, whereas the ASV matrix of the HFD group showed a non-significant positive correlation with this parameter. In addition, marked differential correlations with ASV composition were observed between the HFD + B6 and HFD groups in terms of serum TCH, TNF-α, and IL-10 concentrations and hepatic IL-1β expression. Specifically, the HFD + B6 group exhibited negative correlations with serum TCH levels, serum TNF-α levels, and hepatic IL-1β expression and a positive correlation with serum IL-10 concentrations. In contrast, the HFD group showed opposite correlation patterns with the aforementioned biomarkers (**Figure 6A**). Spearman’s rank correlation analysis was conducted among the abundance of the 10 key discriminatory bacterial ASVs, serum biomarkers, and 12 significantly altered lipids and lipid-like metabolites. The results demonstrated that S24-7_ASV76453, *Lactobacillus*_ASV50156, and *Akkermansia*_ASV10889, all of which had higher relative abundances in the HFD + B6 group, were positively correlated with IL-10, oleic acid, linoleic acid, and lipoxin A4 levels. Conversely, the other discriminatory bacterial ASVs that predominated in the HFD group displayed opposite correlation trends (**Figures 6B–D**). Overall, these correlation analyses indicate that the ASVs significantly enriched by *L. rhamnosus* B6 intervention are closely associated with the core metabolic and inflammatory biomarkers, suggesting that the *L. rhamnosus* B6-modulated gut microbiota may play a crucial role in mediating the ameliorative effects on MAFLD.

## 4 Discussion

The incidence of metabolic dysfunction-associated fatty liver disease (MAFLD) is soaring, and it has emerged as a global burden that severely endangers human health (27). Although the underlying mechanisms by which the gut–liver axis contributes to the development of MAFLD remain largely elusive, the roles of gut microbiota in the progression of MAFLD are gradually being unraveled (28). Research on probiotics improving MAFLD via the gut–liver axis has become an *ad hoc* topic (29–31). In this study, the colonization capacity of *L. rhamnosus* B6 was determined, and its comprehensive effect on alleviating MAFLD was elucidated using 16S metagenomics combined with untargeted metabolomics. Specifically, *L. rhamnosus* B6 mitigates MAFLD by reducing LPS-induced gut inflammation and regulating lipid metabolism by modulating the oxidation pathways of long-chain saturated fatty acids and the levels of unsaturated fatty acids and their derivatives.

The health-promoting benefits of probiotics are closely associated with their ability to survive transit through the digestive tract, occupy ecological niches, and colonize the host intestinal mucosa (32, 33). However, many studies investigating the gut colonization of individual probiotics have relied on fecal microbiome composition, which only partially correlates with the local mucosal microbiome (34). In our study, we evaluated the *in vivo* colonization capacity of *L. rhamnosus* B6 by analyzing the microbiome composition of the mouse intestinal mucosa and contents rather than feces. The results demonstrated that an OTU with the same 16S V3–V4 sequence of *L. rhamnosus* accounted for a certain proportion of the gut contents and mucosa (**Figures 1E, F**), indicating the colonization capacity of *L. rhamnosus* B6, an essential prerequisite for its role in alleviating MAFLD.

The etiology of MAFLD is complicated, and accumulating evidence suggests that intestinal flora dysbiosis induced by a long-term high-fat diet increases pro-inflammatory substrate levels, which is a pivotal factor contributing to MAFLD (35–37). Previous studies have highlighted that overgrowth of Desulfovibrionaceae and Enterobacteriaceae in the gut produces excessive LPS (38–40), which can be recognized by Toll-like receptor-4 (TLR4) and activate pro-inflammatory signaling pathways, thereby promoting the chronic inflammatory state of MAFLD (41, 42). In the present study, *L. rhamnosus* B6 intervention downregulated the intestinal LPS synthesis pathway by reducing the abundance of Desulfovibrionaceae and Enterobacteriaceae reduce (**Figure 4C**), decreased serum levels of pro-inflammatory cytokine (TNF-α and IL-1β), enhanced anti-inflammatory cytokine (IL-10) levels, and decreased the hepatic expression of pro-inflammatory cytokine (TNF-α and IL-1β) and NF-κB, which subsequently alleviated liver inflammation (**Figure 3**). The inhibitory effects of *L. rhamnosus* B6 intervention on intestinal LPS synthesis was partially validated by its *in vitro* antagonistic activity against two notorious LPS-producing bacteria, such as *E*. *coli* and *S*. *enterica* (**Supplementary Figure S1**). Interestingly, *L. rhamnosus* B6 administration also reduced the abundance of *Cupriavidus* and the pathways involved in myo-inositol degradation (PWY-7237 and PWY-562), respectively. Myo-inositol deficiency caused by intestinal flora-mediated myo-inositol degradation is closely related to fatty liver in both humans and animals (43), and myo-inositol supplementation can reduce hepatic triglyceride and cholesterol accumulation (44, 45). Therefore, regulating intestinal flora to reduce myo-inositol degradation may contribute to the MAFLD-alleviating effect of *L. rhamnosus* B6.

In addition to inflammation, hepatic triglyceride accumulation is a characteristic feature of MAFLD (46), and the fatty acid β-oxidation pathway is one of the major metabolic pathways that determines hepatic triglyceride content (47). *L. rhamnosus* B6 treatment upregulated the mitochondrial β-oxidation pathway of long-chain saturated fatty acids (**Figure 5F**) and significantly reduced hepatic triglyceride levels (**Figure 2E**). Consistent with this, *L. rhamnosus* B6 intervention significantly improved the levels of unsaturated fatty acids (UFAs) and their derivatives, including oleic acid, linoleic acid, and lipoxin A4, and upregulated α-linolenic and linoleic acid metabolism (**Figures 5E, F**). UFAs are widely recognized for their beneficial effects on lipid metabolism in patients with MAFLD. Oleic acid has been reported to ameliorate hepatocellular lipotoxicity in previous studies (48, 49). As an omega-6 UFA, linoleic acid exerts a lipid-lowering effect (50). Depletion of omega-6 UFAs in the serum of NAFLD-HCC patients indicates that linoleic acid may play an important role in improving NAFLD (51). Lipoxin A4 (LXA4), a bioactive lipid mediator derived from omega-6 UFAs, exhibited anti-inflammatory effects and therapeutic benefits in *in vitro* liver fibrosis models (52). Additionally, α-linoleic acid and linoleic acid metabolism has been reported to be associated with metabolic syndrome in NAFLD rats (50). Furthermore, the negative correlation between hepatic TG levels and the lipid molecules elevated by *L. rhamnosus* B6 (including oleic acid, linoleic acid, and lipoxin A4) (**Figure 6D**) suggests that *L. rhamnosus* B6 may also regulate lipid metabolism by increasing UFA levels, which is critical for maintaining energy homeostasis, thereby ameliorating MAFLD (53).

In addition to fatty acids, sphingolipid metabolism is closely associated with MAFLD development. The liver is a key site for ceramide synthesis. Ceramide, as the central intermediate in sphingolipid metabolism, and an imbalanced ratio of sphingolipid species can promote weight gain and liver inflammation (54). In the present study, ceramide (d18:1/18:2), which exhibited a positive correlation with pro-inflammatory cytokine expression (**Figure 6D**), was significantly reduced by *L. rhamnosus* B6 intervention (**Figure 5E**). Our results are consistent with those of previous studies demonstrating that increased ceramide levels are closely associated with hepatic steatosis (55). Moreover, the phenylalanine and tyrosine metabolism pathways were also regulated by *L. rhamnosus* B6. Although few studies have focused on the relationship between phenylalanine and tyrosine metabolism and MAFLD development, it has been reported that phenylalanine and tyrosine can serve as metabolic signatures of hepatotoxicity (56), and these metabolites were found to be significantly decreased in patients with MAFLD (57).

Compared with other *L. rhamnosus* strains, although these strains have also been reported to alleviate liver diseases by modulating the gut microbiota, their inhibitory effects on harmful intestinal bacteria are mostly reflected in the 16S rRNA gene sequencing results of fecal samples and have not been validated *in vitro* (13, 15–17). Moreover, previous studies have mainly focused on short-chain fatty acid profiles and hepatic carbohydrate metabolism (16, 17). However, our results provide new insights into the alleviatory effects of *L. rhamnosus* B6 on MAFLD. First, we used intestinal contents as the research object to explore the impact of *L. rhamnosus* B6 on the gut microbiome, which can more accurately reflect the impact of the administered probiotics on the microbial community. Second, except for unraveling the role of *L. rhamnosus* in improving MAFLD by reducing the inflammatory reaction induced by LPS, we are the first to uncover a novel pathway through which probiotics alleviate MAFLD: increasing myo-inositol levels by inhibiting potential myo-inositol-degrading gut microbes.

However, the present study had certain limitations. First, the detection of myo-inositol and UFA levels following *L. rhamnosus* B6 supplementation was insufficient. The identity of key myo-inositol-degrading microorganisms involved in MAFLD pathogenesis and the antagonistic activity of *L. rhamnosus* B6 against these degraders remain to be verified. Furthermore, the number of biological replicates in untargeted metabolomics experiments (six samples per group) was relatively small, which might have exerted a modest impact on the results related to subtle metabolic correlations.

## 5 Conclusions

In conclusion, our study demonstrated that *L. rhamnosus* B6 supplementation effectively ameliorates MAFLD by reducing hepatic steatosis and inflammation. These effects may be attributed to its inhibitory activity against LPS-producer and myo-inositol-degrading bacteria in the gut, as well as its ability to regulate lipid metabolism by enhancing UFA levels. Collectively, our results suggest that *L. rhamnosus* B6 is an effective probiotic for ameliorating MAFLD in mice.

## Data Availability

The datasets presented in this study can be found in online repositories. The names of the repository/repositories and accession number(s) can be found in the article/**Supplementary Material**. The genome sequence of Lacticaseibacillus rhamnosus B6 is available in the SRA under accession no. PRJNA687329. The 16S rRNA data were deposited in the SRA under BioProject accession nos. PRJNA1070373 and PRJNA1130061.

## Supplementary material

Supplementary Table 1 Primer sequences of RT-qPCR.

Supplementary Table 2 Detailed information on differentially expressed lipid and lipid-like molecules.

Supplementary Figure 1 Inhibition zones of *L. rhamnosus* B6 fermentation supernatant against indicator Enterobacteriaceae strains.

## References

[1] Eslam M Newsome PN Sarin SK Anstee QM Targher G Romero-Gomez M . A new definition for metabolic dysfunction-associated fatty liver disease: An international expert consensus statement. J Hepatol. (2020) 73:202–9. doi: 10.1016/j.jhep.2020.03.039, PMID: 32278004

[2] Eslam M Sanyal AJ George J . International consensus panel. MAFLD: A consensus-driven proposed nomenclature for metabolic associated fatty liver disease. Gastroenterology. (2020) 158:1999–2014.e1. doi: 10.1053/j.gastro.2019.11.312, PMID: 32044314

[3] Eslam M El-Serag HB Francque S Sarin SK Wei L Bugianesi E . Metabolic (dysfunction)-associated fatty liver disease in individuals of normal weight. Nat Rev Gastroenterol Hepatol. (2022) 19:638–51. doi: 10.1038/s41575-022-00635-5, PMID: 35710982

[4] Pan X Wen SW Kaminga AC Liu A . Gut metabolites and inflammation factors in non-alcoholic fatty liver disease: A systematic review and meta-analysis. Sci Rep. (2020) 10:8848. doi: 10.1038/s41598-020-65051-8, PMID: 32483129 PMC7264254

[5] Buzzetti E Pinzani M Tsochatzis EA . The multiple-hit pathogenesis of non-alcoholic fatty liver disease (NAFLD). Metabolism. (2016) 65:1038–48. doi: 10.1016/j.metabol.2015.12.012, PMID: 26823198

[6] Arai N Miura K Aizawa K Sekiya M Nagayama M Sakamoto H . Probiotics suppress nonalcoholic steatohepatitis and carcinogenesis progression in hepatocyte-specific PTEN knockout mice. Sci Rep. (2022) 12:16206. doi: 10.1038/s41598-022-20296-3, PMID: 36171333 PMC9519992

[7] Wang H Mehal W Nagy LE Rotman Y . Immunological mechanisms and therapeutic targets of fatty liver diseases. Cell Mol Immunol. (2021) 18:73–91. doi: 10.1038/s41423-020-00579-3, PMID: 33268887 PMC7852578

[8] Wang Q Wang Z Pang B Zheng H Cao Z Feng C . Probiotics for the improvement of metabolic profiles in patients with metabolic-associated fatty liver disease: A systematic review and meta-analysis of randomized controlled trials. Front Endocrinol. (2022) 13:1014670. doi: 10.3389/fendo.2022.1014670, PMID: 36407321 PMC9670148

[9] Lee NY Shin MJ Youn GS Yoon SJ Choi YR Kim HS . *Lactobacillus* attenuates progression of nonalcoholic fatty liver disease by lowering cholesterol and steatosis. Clin Mol Hepatol. (2021) 27:110–24. doi: 10.3350/cmh.2020.0125, PMID: 33317254 PMC7820205

[10] Lin JH Lin CH Kuo YW Liao CA Chen JF Tsai SY . Probiotic *Lactobacillus fermentum* TSF331, *Lactobacillus reuteri TSR332*, and *Lactobacillus plantarum* TSP05 improved liver function and uric acid management-A pilot study. PLoS One. (2024) 19:e0307181. doi: 10.1371/journal.pone.0307181, PMID: 39046973 PMC11268587

[11] Ahn SB Jun DW Kang BK Lim JH Lim S Chung MJ . Randomized, double-blind, placebo-controlled study of a multispecies probiotic mixture in nonalcoholic fatty liver disease. Sci Rep. (2019) 9:5688. doi: 10.1038/s41598-019-42059-3, PMID: 30952918 PMC6450966

[12] Jang HR Park HJ Kang D Chung H Nam MH Lee Y . A protective mechanism of probiotic *Lactobacillus* against hepatic steatosis via reducing host intestinal fatty acid absorption. Exp Mol Med. (2019) 51:1–14. doi: 10.1038/s12276-019-0293-4, PMID: 31409765 PMC6802638

[13] Bajaj JS Heuman DM Hylemon PB Sanyal AJ Puri P Sterling RK . Randomised clinical trial: *Lactobacillus GG* modulates gut microbiome, metabolome and endotoxemia in patients with cirrhosis. Aliment Pharmacol Ther. (2014) 39:1113–25. doi: 10.1111/apt.12695, PMID: 24628464 PMC3989370

[14] Kim B Park KY Ji Y Park S Holzapfel W Hyun CK . Protective effects of *Lactobacillus rhamnosus* GG against dyslipidemia in high-fat diet-induced obese mice. Biochem Biophys Res Commun. (2016) 473:530–6. doi: 10.1016/j.bbrc.2016.03.107, PMID: 27018382

[15] Wang SL Liang S Li SY Fu JW Wang ZY Zhu DQ . *Lactobacillus rhamnosus* GG attenuates MASLD/MASH progression by modulating gut microbiota and metabolic pathways. Front Microbiol. (2025) 16:1586678. doi: 10.3389/fmicb.2025.1586678, PMID: 40778202 PMC12328336

[16] Liao YT Huang J Lin BR Liang JT Huang KW . *Lactobacillus rhamnosus* ameliorates dyslipidemia and liver steatosis in a rat model fed high-fat diet. Gastroenterol Res Pract. (2025) 2025:5540686. doi: 10.1155/grp/5540686, PMID: 41122631 PMC12537184

[17] Sun M Wu T Zhang G Liu R Sui W Zhang M . *Lactobacillus rhamnosus* LRa05 improves lipid accumulation in mice fed with a high fat diet via regulating the intestinal microbiota, reducing glucose content and promoting liver carbohydrate metabolism. Food Funct. (2020) 11:9514–25. doi: 10.1039/d0fo01720e, PMID: 33063800

[18] Han J Xia W Wang D Wang Y Liu Z Wu Z . Characterization of an exopolysaccharide synthesized by *Lacticaseibacillus rhamnosus* B6 and its immunomodulatory activity *in vitro*. Int J Biol Macromol. (2024) 264:130576. doi: 10.1016/j.ijbiomac.2024.130576, PMID: 38442828

[19] Zhang X Zheng Y Liu Z Su M Wu Z Xu X . Integrated analysis of characteristic volatile flavor formation mechanisms in probiotic co-fermented cheese by untargeted metabolomics and sensory predictive modeling. Food Res Int. (2025) 211:116379. doi: 10.1016/j.foodres.2025.116379, PMID: 40356103

[20] Zhang X Zheng Y Liu Z Su M Wu Z Zhang H . Insights into characteristic metabolites and potential bioactive peptides profiles of fresh cheese fermented with three novel probiotics based metabolomics and peptidomics. Food Chem X. (2024) 21:101147. doi: 10.1016/j.fochx.2024.101147, PMID: 38312486 PMC10837474

[21] Yang T Zhang J Zhang R Zhang J Liu Z Wu Z . Cold stress enhances cryotolerance in *Lacticaseibacillus rhamnosus* B6 via membrane lipid remodeling and differential protein expression. Curr Res Microb Sci. (2025) 9:100453. doi: 10.1016/j.crmicr.2025.100453, PMID: 40837524 PMC12363473

[22] Smith CA Want EJ O’Maille G Abagyan R Siuzdak G . XCMS: processing mass spectrometry data for metabolite profiling using nonlinear peak alignment, matching, and identification. Anal Chem. (2006) 78:779–87. doi: 10.1021/ac051437y, PMID: 16448051

[23] Louail P Rainer J . LC-MS data preprocessing and analysis with xcms (2025). Available online at: https://sneumann.github.io/xcms/articles/xcms.html (Accessed October 13, 2025).

[24] Luo K Chen Y Fang S Wang S Wu Z Li H . Study on inflammation and fibrogenesis in MAFLD from 2000 to 2022: a bibliometric analysis. Front Endocrinol. (2023) 14:1231520. doi: 10.3389/fendo.2023.1231520, PMID: 37720529 PMC10500306

[25] Schuster S Cabrera D Arrese M Feldstein AE . Triggering and resolution of inflammation in NASH. Nat Rev Gastroenterol Hepatol. (2016) 15:349–64. doi: 10.1038/s41575-018-0009-6, PMID: 29740166

[26] Badmus OO Hillhouse SA Anderson CD Hinds TD Stec DE . Molecular mechanisms of metabolic associated fatty liver disease (MAFLD): functional analysis of lipid metabolism pathways. Clin Sci. (2022) 136:1347–66. doi: 10.1042/CS20220572, PMID: 36148775 PMC9508552

[27] Huang W Shen B Li X Zhang T Zhou X . Benefits of combining *sonchus brachyotus DC.* Extracts and synbiotics in alleviating non-alcoholic fatty liver disease. Foods. (2023) 12:3393. doi: 10.3390/foods12183393, PMID: 37761102 PMC10530047

[28] Albillos A de Gottardi A Rescigno M . The gut-liver axis in liver disease: Pathophysiological basis for therapy. J Hepatol. (2020) 72:558–77. doi: 10.1016/j.jhep.2019.10.003, PMID: 31622696

[29] Zhao Z Chen L Zhao Y Wang C Duan C Yang G . *Lactobacillus plantarum* NA136 ameliorates nonalcoholic fatty liver disease by modulating gut microbiota, improving intestinal barrier integrity, and attenuating inflammation. Appl Microbiol Biotechnol. (2020) 104:5273–82. doi: 10.1007/s00253-020-10633-9, PMID: 32335723

[30] Kim DY Park JY Gee HY . *Lactobacillus plantarum* ameliorates NASH-related inflammation by upregulating L-arginine production. Exp Mol Med. (2023) 55:2332–45. doi: 10.1038/s12276-023-01102-0, PMID: 37907736 PMC10689779

[31] Luo M Yan J Wu L Wu J Chen Z Jiang J . Probiotics alleviated nonalcoholic fatty liver disease in high-fat diet-fed rats via gut microbiota/FXR/FGF15 signaling pathway. J Immunol Res. (2021) 2021:2264737. doi: 10.1155/2021/2264737, PMID: 34458376 PMC8387197

[32] Han S Lu Y Xie J Fei Y Zheng G Wang Z . Probiotic gastrointestinal transit and colonization after oral administration: A long journey. Front Cell Infect Microbiol. (2021) 11:609722. doi: 10.3389/fcimb.2021.609722, PMID: 33791234 PMC8006270

[33] Yao M Xie J Du H McClements DJ Xiao H Li L . Progress in microencapsulation of probiotics: A review. Compr Rev Food Sci Food Saf. (2020) 19:857–74. doi: 10.1111/1541-4337.12532, PMID: 33325164

[34] Zmora N Zilberman-Schapira G Suez J Mor U Dori-Bachash M Bashiardes S . Personalized gut mucosal colonization resistance to empiric probiotics is associated with unique host and microbiome features. Cell. (2018) 174:1388–405. doi: 10.1016/j.cell.2018.08.041, PMID: 30193112

[35] Plaza-Díaz J Solís-Urra P Rodríguez-Rodríguez F Olivares-Arancibia J Navarro-Oliveros M Abadía-Molina F . The gut barrier, intestinal microbiota, and liver disease: molecular mechanisms and strategies to manage. Int J Mol Sci. (2020) 21:8351. doi: 10.3390/ijms21218351, PMID: 33171747 PMC7664383

[36] Portincasa P Bonfrate L Khalil M Angelis M Calabrese FM D’Amato M . Intestinal barrier and permeability in health. Biomedicines. (2021) 10:83. doi: 10.3390/biomedicines10010083, PMID: 35052763 PMC8773010

[37] An L Wirth U Koch D Schirren M Drefs M Koliogiannis D . The role of gut-derived lipopolysaccharides and the intestinal barrier in fatty liver diseases. J Gastrointest Surg. (2022) 26:671–83. doi: 10.1007/s11605-021-05188-7, PMID: 34734369 PMC8926958

[38] Lin TL Shu CC Chen YM Lu JJ Wu TS Lai WF . Like cures like: pharmacological activity of anti-inflammatory lipopolysaccharides from gut microbiome. Front Pharmacol. (2020) 11:554. doi: 10.3389/fphar.2020.00554, PMID: 32425790 PMC7212368

[39] Chen Z Lv M Liang J Yang K Li F Zhou Z . Neuropeptide Y-mediated gut microbiota alterations aggravate postmenopausal osteoporosis. Adv Sci. (2023) 10:e2303015. doi: 10.1002/advs.202303015, PMID: 37857552 PMC10667841

[40] Mohammad S Thiemermann C . Role of metabolic endotoxemia in systemic inflammation and potential interventions. Front Immunol. (2021) 11:594150. doi: 10.3389/fimmu.2020.594150, PMID: 33505393 PMC7829348

[41] Villard A Boursier J Andriantsitohaina R . Bacterial and eukaryotic extracellular vesicles and nonalcoholic fatty liver disease: new players in the gut-liver axis? Am J Physiol Gastrointest Liver Physiol. (2021) 320:G485–95. doi: 10.1152/ajpgi.00362.2020, PMID: 33471632

[42] Ren G Bai C Yi S Cong Q Zhu Y . Mechanisms and therapeutic strategies for MAFLD targeting TLR4 signaling pathways. J Innate Immun. (2024) 16:45–55. doi: 10.1159/000535524, PMID: 38128497 PMC10783892

[43] Caputo M Bona E Leone I Samà MT Nuzzo A Ferrero A . Inositols and metabolic disorders: From farm to bedside. J Tradit Complement Med. (2020) 10:252–9. doi: 10.1016/j.jtcme.2020.03.005, PMID: 32670820 PMC7340869

[44] Zhou J Zhang Q Zhao Y Zou Y Chen M Zhou S . The relationship of *Megamonas* species with nonalcoholic fatty liver disease in children and adolescents revealed by metagenomics of gut microbiota. Sci Rep. (2022) 12:22001. doi: 10.1038/s41598-022-25140-2, PMID: 36539432 PMC9767906

[45] Pani A Giossi R Menichelli D Fittipaldo VA Agnelli F Inglese E . Inositol and non-alcoholic fatty liver disease: A systematic review on deficiencies and supplementation. Nutrients. (2020) 12:3379. doi: 10.3390/nu12113379, PMID: 33153126 PMC7694137

[46] Semova I Biddinger SB . Triglycerides in nonalcoholic fatty liver disease: guilty until proven innocent. Trends Pharmacol Sci. (2021) 42:183–90. doi: 10.1016/j.tips.2020.12.001, PMID: 33468321 PMC10065162

[47] Hodson L Gunn PJ . The regulation of hepatic fatty acid synthesis and partitioning: the effect of nutritional state. Nat Rev Endocrinol. (2019) 15:689–700. doi: 10.1038/s41574-019-0256-9, PMID: 31554932

[48] Liu X Li X Su S Yuan Y Liu W Zhu M . Oleic acid improves hepatic lipotoxicity injury by alleviating autophagy dysfunction. Exp Cell Res. (2023) 429:113655. doi: 10.1016/j.yexcr.2023.113655, PMID: 37253404

[49] Zeng X Zhu M Liu X Chen X Yuan Y Li L . Oleic acid ameliorates palmitic acid induced hepatocellular lipotoxicity by inhibition of ER stress and pyroptosis. Nutr Metab. (2020) 17:11. doi: 10.1186/s12986-020-0434-8, PMID: 32021639 PMC6990600

[50] Cui H Li Y Cao M Liao J Liu X Miao J . Untargeted metabolomic analysis of the effects and mechanism of nuciferine treatment on rats with nonalcoholic fatty liver disease. Front Pharmacol. (2020) 11:858. doi: 10.3389/fphar.2020.00858, PMID: 32581811 PMC7295953

[51] Lewinska M Santos-Laso A Arretxe E Alonso C Zhuravleva E Jimenez-Agüero R . The altered serum lipidome and its diagnostic potential for Non-Alcoholic Fatty Liver (NAFL)-associated hepatocellular carcinoma. EBioMedicine. (2021) 73:103661. doi: 10.1016/j.ebiom.2021.103661, PMID: 34740106 PMC8577325

[52] Kurtoğlu EL Kayhan B Gül M Kayhan B Akdoğan Kayhan M Karaca ZM . A bioactive product lipoxin A4 attenuates liver fibrosis in an experimental model by regulating immune response and modulating the expression of regeneration genes. Turk J Gastroenterol. (2019) 30:745–57. doi: 10.5152/tjg.2019.18276, PMID: 31418419 PMC6699576

[53] Sun Q Xing X Wang H Wan K Fan R Liu C . SCD1 is the critical signaling hub to mediate metabolic diseases: Mechanism and the development of its inhibitors. BioMed Pharmacother. (2024) 170:115586. doi: 10.1016/j.biopha.2023.115586, PMID: 38042113

[54] Régnier M Polizzi A Guillou H Loiseau N . Sphingolipid metabolism in non-alcoholic fatty liver diseases. Biochimie. (2019) 159:9–22. doi: 10.1016/j.biochi.2018.07.021, PMID: 30071259

[55] Simon J Ouro A Ala-Ibanibo L Presa N Delgado TC Martínez-Chantar ML . Sphingolipids in non-alcoholic fatty liver disease and hepatocellular carcinoma: ceramide turnover. Int J Mol Sci. (2019) 21:40. doi: 10.3390/ijms21010040, PMID: 31861664 PMC6982102

[56] Liu P Wu J Yu X Guo L Zhao L Ban T . Metabolomics and network analyses reveal phenylalanine and tyrosine as signatures of anthracycline-induced hepatotoxicity. Pharmaceuticals. (2023) 16:797. doi: 10.3390/ph16060797, PMID: 37375744 PMC10302024

[57] Masoodi M Gastaldelli A Hyötyläinen T Arretxe E Alonso C Gaggini M . Metabolomics and lipidomics in NAFLD: biomarkers and non-invasive diagnostic tests. Nat Rev Gastroenterol Hepatol. (2021) 18:835–56. doi: 10.1038/s41575-021-00502-9, PMID: 34508238

