# Supplementary figures and images for: Lacticaseibacillus rhamnosus B6 alleviates metabolic dysfunction-associated fatty liver disease by suppressing intestinal LPS synthesis and regulating lipid metabolism
Source: Front Endocrinol (Lausanne). 2026 Feb 16;17:1755982. doi: 10.3389/fendo.2026.1755982 (PMC12950561; doi:10.3389/fendo.2026.1755982)

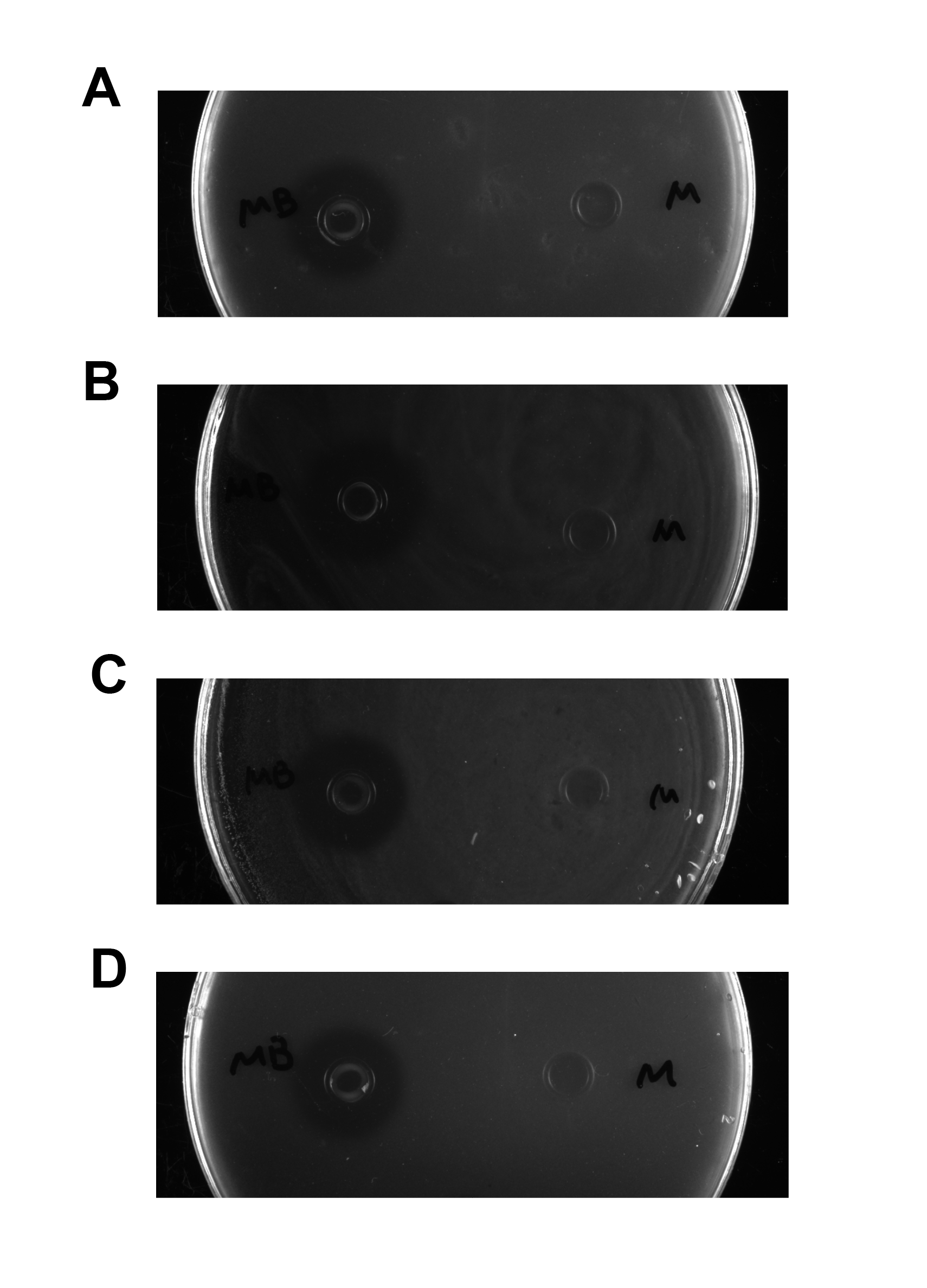

Supplement: Supplementary Table 1 — Primer sequences of RT-qPCR. [file DataSheet1.zip › S1-01.tif]
